# A Blended Artificial Intelligence Approach for Spectral Classification of Stars in Massive Astronomical Surveys

**DOI:** 10.3390/e22050518

**Published:** 2020-05-01

**Authors:** Carlos Dafonte, Alejandra Rodríguez, Minia Manteiga, Ángel Gómez, Bernardino Arcay

**Affiliations:** 1 CITIC—Department of Computer Science and IT, University of A Coruna, 15071 A Coruña, Spain; alejandra.rodriguez@udc.es (A.R.); angel.gomez@udc.es (Á.G.); bernardino.arcay@udc.es (B.A.); 2 CITIC—Department of Navigation and Earth Sciences, University of A Coruna, 15071 A Coruña, Spain; minia.manteiga@udc.es

**Keywords:** hybrid systems, MK classification, spectral features, astronomical databases, artificial neural networks

## Abstract

This paper analyzes and compares the sensitivity and suitability of several artificial intelligence techniques applied to the Morgan–Keenan (MK) system for the classification of stars. The MK system is based on a sequence of spectral prototypes that allows classifying stars according to their effective temperature and luminosity through the study of their optical stellar spectra. Here, we include the method description and the results achieved by the different intelligent models developed thus far in our ongoing stellar classification project: fuzzy knowledge-based systems, backpropagation, radial basis function (RBF) and Kohonen artificial neural networks. Since one of today’s major challenges in this area of astrophysics is the exploitation of large terrestrial and space databases, we propose a final hybrid system that integrates the best intelligent techniques, automatically collects the most important spectral features, and determines the spectral type and luminosity level of the stars according to the MK standard system. This hybrid approach truly emulates the behavior of human experts in this area, resulting in higher success rates than any of the individual implemented techniques. In the final classification system, the most suitable methods are selected for each individual spectrum, which implies a remarkable contribution to the automatic classification process.

## 1. Introduction

Today’s astrophysicists are frequently dealing with the analysis of complex data from one or more astronomical surveys, which typically contain millions or even hundreds of millions of sources, from which they want to determine attributes such as their membership to a given class of astronomical objects (star, galaxy, and quasar), or their main physical parameters. To analyze the information in these enormous volumes of data, it is necessary to resort to automatic processing techniques. Many of these files are open to the international scientific community for study, but their analysis is a challenge for astronomers of the 21st century, since it requires mastery of advanced computing techniques, based on statistics and the use of methodologies such as those derived from Artificial Intelligence (AI), in what has come to be called “data mining in astronomy”. In this paper, we present the application of a variety of AI-based techniques to the retrieval of information present in stellar spectra obtained from telescopes, with the goal of providing a reliable hybrid system that makes it easier for the astronomer to classify stars in the MK system, a standard in stellar astrophysics.

The radiation spectrum of a star (i.e., the energy distribution as a function of the wavelength) is a black body-like curve that shows the temperature of the outer regions of stars, their photosphere, while the occurrence of spectral lines and bands in the light distribution is a consequence of the energy transitions of the elements and molecules that compose the stellar plasma. The relative intensities of these spectral characteristics are strongly dependent on the physical features (temperature, pressure, etc.) and on the presence and quantities of chemical elements in the stellar atmosphere, in such a way that stellar spectroscopy has become one of the most important tools to study those properties in stars.

In the 1880s, Williamina P. Fleming, Antonia C. Maury, and Ann Jump Cannon, among others, developed a pioneering work of massive classification of stellar spectra: The Henry Draper catalog of stars [1]. Stellar types were originally arranged in alphabetic order, beginning at A for stars with the strongest hydrogen lines. Soon it became clear that the spectral types were related to the star temperature, thus the final series was obtained after some subsequent modifications: O, B, A, F, G, K, and M, from hot to cool stars (additional letters were used to designate nova and less common types of stars). Numbers 0–9 are used to subdivide the types, applying the higher numbers to cooler stars. The hottest stars are known as early stars and the coldest as late stars. This scheme was created at the Harvard College Observatory, thus it was called the Harvard stellar classification system.

In 1940, the American astronomers Morgan, Keenan, and Kellman carried out an expansion of the Harvard sequence to include luminosity [2]. In the so-called MKK, or simply MK, system (following the authors’ names or first two authors’ names) a roman numeral is appended to the Harvard type to indicate the luminosity class of the star: I for super-giants, II for bright giants, III for giants, IV for sub-giants, and V for dwarfs or main sequence stars. The different levels of luminosity refer to the absolute magnitude of the star, which is a measure of its luminosity on a logarithmic scale with a negative factor, whereby smaller magnitudes correspond to brighter stars.

Human experts often classify stellar spectra with the support of a guiding catalog of prototypical spectra with a reliable classification in the MK system, which have been previously selected to be used as a complete reference in the manual classification process. The classical spectral classification process mainly focuses on the substantial information provided by certain lines and spectral areas, so that, to directly compare the non-classified stars with those of the reference catalog, it is necessary to adapt all the spectra to the same scale and then normalize them and isolate their continuous component (affected by interstellar reddening).

Once human classifiers have scaled and normalized target spectra, they try to guess their spectral type and luminosity in the MK system. The morphological differences between spectral types are found in the intensity of the absorption lines of hydrogen and helium, and in the presence or absence of certain metals and molecular bands (Ca, Mg, Fe, C, Ti, etc. bands). However, the luminosity is related to the width of some specific lines in the spectrum. Therefore, the expert classifiers measure and analyze the relationship between some absorption lines (H, He, Ca, etc.) and the depth of certain relevant molecular bands (TiO, CH, etc.), obtaining an initial classification that usually includes a first approximation to the spectral type and luminosity class [3]. This tentative classification is completed by assigning the spectral subtype that best matches when the unclassified spectra and the catalog templates are superposed. Sometimes it is impossible to decide which spectral subtype a star belongs to, so that they finally adopt a mixed classification (e.g., types B02, F79, etc.). 

The described manual classification technique is quite subjective and strongly depends on the criteria and experience of the expert classifiers. Moreover, their practical application is often not feasible, especially when the number of spectra is very high, as it would require a great deal of time and human resources. In fact, nowadays it is possible to collect spectral information of several hundreds of stars in one night of telescope observation, using modern techniques of multi-object spectroscopy, or many more objects in the case of spatial missions. In these situations, the classical manual technique is no longer operative, and it would be desirable to replace it with automatic and non-subjective classification schemes. 

In previous works, we described an initial knowledge-based system for the classification of the low-resolution optical spectra of super-giant, giant, and dwarf stars [4]. Then, we refined this system with a first hybrid approach based on the combination of the most effective neural networks [5]. The obtained results encouraged us to adapt such a system to manage stars belonging to other evolutionary stages (with different luminosity classes), while adding new intelligent techniques, such as Takagi–Sugeno fuzzy reasoning, to complete the MK computerized classification process. 

Certain well-known previous works have also applied AI techniques and deep learning to the stellar classification field [6,7,8,9,10,11,12], obtaining different performance in the classification. The big data and AI processing on massive catalogs (that do not stop growing in number) go hand in hand. From the first works that explicitly mention the datamining concept in 2000 [13], to publications that propose robust machine learning techniques for this task (also using the MK system) on observations gathered in the Dark Sky observatory [14], they are also currently working with collaborative concepts innovators such as Galaxy Zoo in the LSST survey [15,16] and applying streaming techniques to characterize variable stars in this same survey [17]. In all these cases, and many others, ML techniques such as the one proposed here are the core of the classification or parameterization, using and combining fuzzy logic, artificial neural networks, and rule-based systems.

Of course, it is not our objective to re-test methods that have already proved their suitability, but to determine our best approach for the problem of automatic spectral classification, to design the system while mainly using a first catalog, to optimize and above all validate its operation based on another well-known standard catalog.

Thus, this paper presents the implementation of several intelligent models to carry out an analysis of the sensitivity and the adaptation capability of different AI techniques to the classification of stellar spectra. This exhaustive study aims to integrate all the techniques into a single intelligent system that is able to guide the process and apply the most appropriate classification method for each situation. Considering the subjective human classification process, we believe that the combination of fuzzy expert systems and neural networks in a single intelligent system could be more flexible and suitable than one that uses only a specific AI technique, and might even mean a better adjustment to the peculiarities of stellar classification. Furthermore, we consider that such a system could also be very helpful in the training of new stellar spectroscopists. 

The following section describes the materials and methods that have been used in the development of this research. Firstly, we specify the different groups of spectra that were chosen for the implementation and evaluation of the models, and all the preprocessing stages that are applied to the data before they are presented to the different techniques. Subsequently, we include some of the morphological algorithms that were developed for knowledge-based systems but are used here to extract the set of parameters that characterizes each spectrum, which are then used as an input for most of the intelligent models. After that, we describe in detail the different AI techniques that were implemented, to finally compare their results for the same sets of unclassified spectra. To conclude, we present the option of a hybrid solution that combines the best tested AI techniques.

## 2. Materials and Methods 

### 2.1. Spectral Catalogs

At the beginning of our research, we mainly worked in the study of a large set of observed sources, candidates to be classified as stars in the post-AGB phase of evolution [18]. In this first approach, we chose as templates the spectra of luminosity level I from the reference catalog used at that time in the manual MK classification method [19].

The results of this initial development showed that, to deal with the whole problem of MK classification of stars, it is highly advisable to compile a solid and robust catalog that provides good resolution for all target spectral types and as many classes of luminosity as possible. Consequently, to design and develop intelligent tools for the rationalization of the manual classification process, we have built a digital database that includes a large sample of optical spectra from public online catalogs: The D. Silva and M. Cornell optical spectral library, now extended to all luminosity levels [19], the catalog of observations of G. Jacoby, D. Hunter and C. Christian [20], and the digital spectra collected by A. Pickles [21]. 

We decided to add a broader set of spectra to facilitate the optimal convergence and generalization of the artificial neural networks, since the training patterns sets notably influence the performance of the models. For this purpose, we selected an additional database of 908 spectra obtained with the ELODIE spectrograph at the French observatory in Haute-Provence; these spectra present greater coverage of MK spectral subtypes and luminosity levels for metallicities ([Fe/H]) from −3.0 to 0.8 [22].

Before applying this final database to the design of automatic classification systems, the classification experts who collaborate in this research visually studied, morphologically analyzed, and contrasted the values of the spectral parameters for the four above-mentioned catalogs; for that purpose, they used the information available in public databases such as SIMBAD [23]. In this way, those spectra that did not satisfy our quality criteria were eliminated: a lot of noise, significant gaps in regions with an abundance of classification indices (e.g., some early types from Pickles), very high or very low levels of metallicity (e.g., some spectra from Silva library with values of [Fe/H] inferior to −1), spectra where MK classifications widely differ from one source to another (e.g., several Jacoby’s spectra), and the spectral subtypes and/or luminosity levels with very few representatives (e.g., the entire spectral type O, unfortunately).

Afterwards, the remaining spectra were analyzed by means of statistical clustering techniques, also discarding those that could not be placed into one of the groups obtained with the different implemented algorithms (K-means, ISODATA, etc.). Because of this complete selection process, we obtained a main catalog with 258 spectra from the libraries of Silva (27), Pickles (97) and Jacoby (134), and a secondary catalog with 500 spectra extracted from the Prugniel database.

**Figure 1:**
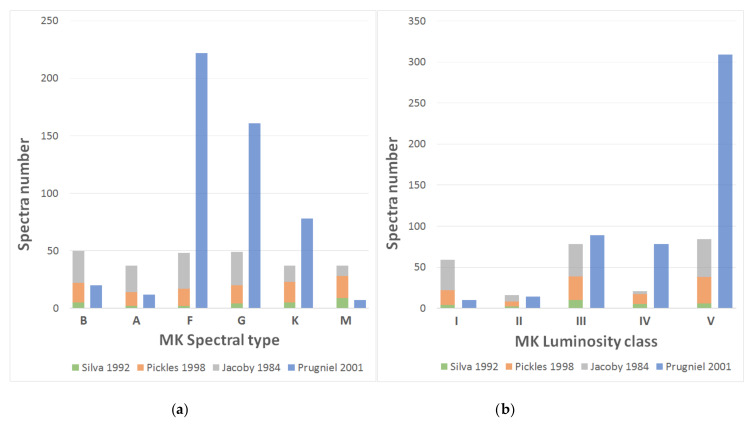
Composition of our definitive reference catalog: (**a**) number of spectra per MK spectral type; and (**b**) number of spectra per MK luminosity class.

Figure 1 shows the final distribution of spectral types and luminosity classes of the reference catalog. The main catalog (Silva, Pickles, and Jacoby) is quite homogeneous in relation to the number of spectra of each type, but it presents some deficiencies in the luminosity classes (II and IV); in the secondary final catalog (Prugniel), most spectra belong to intermediate spectral types (F–K) and to luminosity class V.

All the spectra of our final database were analyzed, obtaining the measurement of some representative parameters such as spectral lines of absorption or molecular bands, so that for each spectral type the typical values were delimited. In most of our implementations, the unclassified spectra that are supplied to the system will be compared with this numerical characterization of the reference spectra; moreover, they are useful to determine the rules of the expert systems as well as to train and validate the neural models. 

More than ten years ago, our research group started working on the Gaia Project, the astrometric cornerstone mission of the European Space Agency (ESA) that was successfully launched and set into orbit in December 2013. Gaia is an astrometric mission that measures parallaxes and movements of the stars in the Milky Way, and also includes a spectrophotometric survey of all objects in the sky up to a visible magnitude of approximately 20.5. The scientific work to prepare the mission archive is organized around the Data Processing and Analysis Consortium (DPAC) where we lead the Outlier Analysis Working Package (WP) and collaborate in others [24,25,26]. In the context of DPAC, we have been testing the performance of different ANNs, both unsupervised and supervised, for classification and parameterization of astrophysical properties of the sources. In particular, we have gained experience in the problem of parameterization of stellar atmospheric properties using the spectra of Gaia RVS instrument [27]. This instrument was mainly built to measure the radial velocity of stars in the near infrared CaII spectral region, but it is also a most helpful tool to estimate the most important stellar APs: effective temperature (Teff), logarithm of surface gravity (log g), abundance of metal elements with respect to hydrogen ([Fe/H]), and abundance of alpha elements with respect to iron ([alpha/Fe]). The results obtained have encouraged us to try those same techniques by applying them to the stellar classification problem in the classic MK system.

### 2.2. Spectral Indices and Sensitivity Analysis

The different types of stars have different observable peculiarities in their spectrum, which are commonly studied to determine the MK classification and also to collect relevant information about stellar properties such us temperature, pressure, density, or radial velocity. Most of these typical spectral features can be isolated by the definition of spectral classification indices [3].

Although there are some classical indices included in the most important classification bibliographical sources, the set of spectral parameters used in this project corresponds to the main features that the spectroscopic experts of our group visually study when trying to obtain the manual classification of the spectra. Most of these spectral indices are grouped into three general types, Intensity (I), Equivalent Width (EW), or Full Width at Half Maximum (FWHM) of absorption and emission spectral lines (He, H, Fe, Ca, K, Mg, etc.); depth (B) of molecular absorption bands (TiO, CH, etc.); and relations between the different measures of the absorption/emission lines (H/He, CaII/ H ratios, etc.). 

Once the set of spectral indices for each particular classification scenario is determined, their values are obtained by applying specific morphological processing algorithms primarily based on the calculation of the molecular bands energy and on the estimation of the local spectral continuum for the absorption/emission lines. These algorithms allow for the extraction of a large number of relevant spectral markers that can be included as classification criteria, although only a small subset of them will be in the final selection of indices. To carry out a study as exhaustive as possible, we initially measured some peculiar parameters that are not normally included in the rules used in the traditional classification process. Likewise, the equivalent width, intensity, and width at half maximum are measured for each spectral line, even when they are not included in the typical classification criteria for a particular context.

Among all the classification criteria collected in the preliminary study of the problem (those indicated by the classification experts as well as those found in the bibliography), a set of 33 morphological features was initially selected. We have contrasted our own classification criteria with those included in the different bibliographic sources, with special emphasis on the study of Lick indices [28].

The suitability of this selected subset of spectral indices was verified by performing a complete sensitivity analysis through the spectra included in the chosen reference catalogs. Thus, the value of each chosen morphological feature was estimated for each spectrum of the reference main catalog (Silva 1992, Pickles 1998, Jacoby 1984); then, these values were ordered so that we can examine their variation with the spectral type and luminosity class. Thanks to this meticulous study, it was possible to establish the real resolution of each index, while delimiting the different types of spectra that it is able to discriminate. 

**Figure 2:**
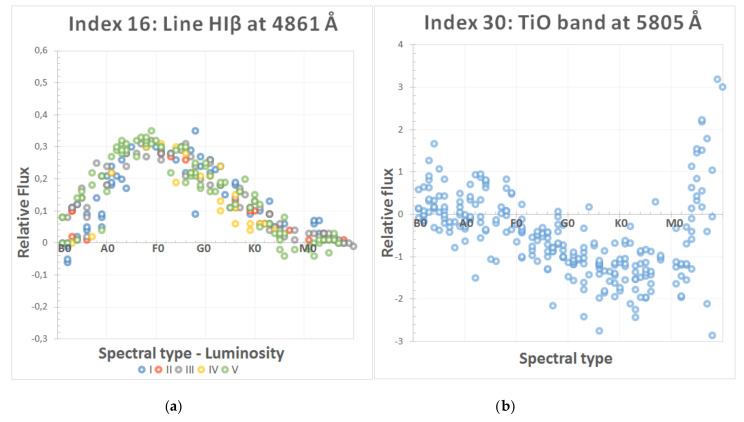
Spectral indices sensitivity analysis: (**a**) hydrogen line at 4861 Å; and (**b**) band of TiO at 5805 Å.

Figure 2 shows two examples of the methodology followed to evaluate the behavior of some of the most significant spectral indices. First, a spectral index (intensity of line Hiβ at 4861 Å) is included in the final selection of parameters for the MK classification because of its excellent performance in the template spectra. Second, we also include an index that was discarded (depth of molecular band TiO at 5805 Å) because its real behavior was not completely congruent with the theoretical one (in fact, it presents similar values for very different spectral types, such as B and M). A more detailed description of this analysis procedure can be found in [29].

Table 1 lists the most relevant results of our sensitivity study, contrasting them with the theoretical classification capability indicated by the experts. Parameters 1–25 constitute the final set of proper indices that are considered to be able to address the MK classification, as they present a clear and reproducible pattern of discrimination between spectral types and/or luminosity classes in the analysis on the 258 spectra from the main reference catalog. The first column contains the definition of each index (an asterisk indicates parameters that are not usually contemplated in the manual classification); the second column shows the types, subtypes, and levels of luminosity that each parameter could potentially discriminate; and the third column specifies the types/classes that it is actually able to delimit, according to the performance achieved in the reference catalogs. The indices highlighted in blue have necessarily been excluded in the processing of the spectra of the secondary database (Prugniel 2001), since they correspond to morphological characteristics that are out of the spectral range of this catalog (4100–6800 Å) and are, therefore, impossible to estimate; unfortunately, this limitation has an unavoidable impact on the performance of some of the implemented techniques.

During the successive tuning processes carried out at this stage, eight indices were excluded from the 33 morphological characteristics of the initial selection. In some cases, as shown in Table 1, the actual found resolution and the theoretical one differed significantly, since they cannot separate the spectral types that, according to experts and bibliographic sources, they should be able to.

The final set of selected spectral indices is included in the classification criteria implemented by means of rules for the expert systems, and in the elaboration of the training and validation patterns for neural models. However, in some specific cases, we have also used certain interesting spectral regions or even the full range of wavelengths for that purpose. Besides, in some implementations, this set of parameters has been reduced by means of another adjustment process based on techniques that allow us to minimize and optimize the number of indices necessary to obtain the MK classification, such as principal component analysis (PCA) or clustering algorithms (K-means, Max-Min, etc.).

### 2.3. Knowledge-Based Systems

In our initial classification project, we designed and developed an automatic system that integrated signal processing, knowledge-based techniques, and a very rudimentary fuzzy logic implementation in order to process the spectra of three luminosity levels (specifically I, III, and V). A complete description of the different versions of this expert system can be found in [4,5,29]. 

In the current phase of our research, we have modified the first expert systems to adapt them to all the spectral types and luminosity levels of the selected spectra of the new reference catalogs. Therefore, the present stellar classifier consists of a knowledge-based system that manages uncertainty and imprecision—characteristic features of human reasoning—by combining traditional production rules, fuzzy logic, and credibility factors. We adapted the methodology of Shortliffe and Buchanan [30] with the development of a fuzzy reasoning scheme by taking the Takagi–Sugeno model [31] as a starting point, and appropriately adapting the Max-Min inference method to include the singularities of the spectral classification process. 

#### Morphological Algorithms

As mentioned in previous sections, the spectral indices are usually defined by the depth of a molecular absorption band (B), the measurement of an absorption/emission line (I, EW, or FWHM), or by the relationships between the different values of the absorption/emission lines. 

A molecular band is a spectral zone where the flux suddenly decreases from the local continuum during a wide wavelength interval. This implies that we can decide whether a molecular band is sufficiently significant by simply measuring its energy. In the present case, after experts have defined the interval for each band by means of the main reference catalog spectra, we calculate the upper threshold line in the limit of that interval by making use of the linear interpolation between the fluxes. Then, we apply a discrete integral to calculate the area between this line and the abscissa axis; and we integrate the flux signal between the band extremes to measure the area around each band. We then subtract the resulting energies and hereby obtain the band flux. When the band deepens and widens, its value turns more negative: this is why positive as well as negative values that are close to zero are not considered bands.

Absorption/emission lines are dark or bright lines that appear in a uniform and continuous spectrum because of a lack (absorption) or excess (emission) of photons in a specific region of wavelengths, in comparison with their closer regions. From a morphological point of view, an absorption line is a descending (ascending for emission) deep peak that appears in an established wavelength zone. The intensity of a line (in erg^−1^cm^−2^s^−1^Å^−1^) is the measurement of its energy flux at the wavelength of the peak with respect to the local spectral continuum of the zone; the equivalent width (usually measured in Å) corresponds to the base of a rectangle whose height is the measurement of the local spectral continuum and has the area of the profile of the line; the width at half maximum (also in Å) will be the width of the line measured at half the maximum intensity. Therefore, it is clearly necessary to calculate the spectral continuum of the surrounding area of each line in order to establish the value of these three parameters.

In the analysis module, we designed an algorithm that estimates the local continuum for each spectral line and obtains a pseudo-continuum that is valid to calculate the value of the spectral parameters of absorption/emission lines (I, EW, and FWHM). During the different development phases of our automatic classification system [4,5,29], the calculation method for this local continuum was modified and adjusted, as the measurements obtained in each option were compared with the actual estimations manually made by the experts.

In the final algorithm, we combined the procedures that proved to be more efficient during the previous implementations with a new method inspired by the technique developed by Cardiel and Gorgas for their INDEX program [32]. Thus, we defined two adjacent intervals to each line (one towards the red and one towards the blue), and then we obtained a value for each lateral continuum by smoothing the flux signal with a low pass filter that excludes the samples with greater standard deviation in each interval (central moving-average of five points). Both estimations (left and right local continuum) are interpolated with polynomial adjustment to obtain the final pseudo-continuum measurement at the wavelength of each line. Through a manual calibration process performed on the spectra belonging to the main catalog, the algorithm established the side intervals, achieving as such a proper adaptation to the peculiarities of each specific absorption/emission line. That is:

(1) $$\left.\begin{array}{c} Cl=\frac{\sum_{j=ll}^{rl}X_{j}\;F\left(\lambda_{j}\right)}{N}\\ Cr=\frac{\sum_{j=ll}^{rl}X_{j}\;F\left(\lambda_{j}\right)}{N} \end{array}\right|X_{j}=\left\{\begin{array}{c} 1,\;\;if\;\sigma \left(\lambda_{j}\right)<\sigma_{n}\;\\ 0,\;\;if\;\sigma \left(\lambda_{j}\right)>\sigma_{n}\; \end{array}\right.,$$

where C*l* and C*r* are the partial estimations of the continuum in the selected range to the left and to the right of the sample where the peak has been detected (λ_p_); *F (λ_j_)* is the flux in sample *j*; *N* the number of samples used in the computation of the left and right partial continuum; *X* is a binary vector that indicates the representative fluxes of the local continuum in the zone; $\sigma \left(\lambda_{j}\right)$ is the local standard deviation in sample *j*; and $\sigma_{n}$ is a standard deviation threshold used to decide if the sample *j* really represents the local continuum and not another morphological feature in that area (band, absorption line, etc.).

**Figure 3:**
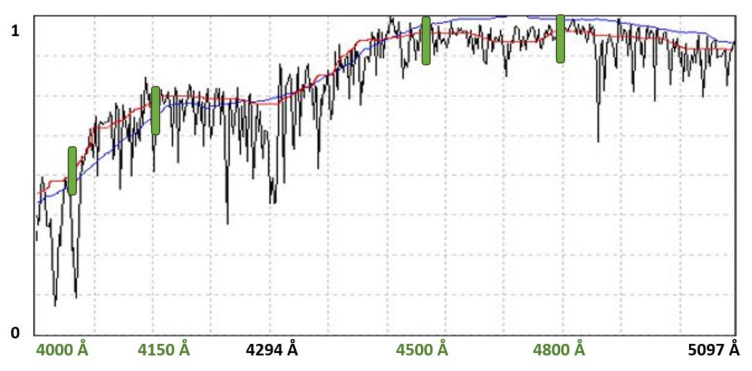
Continuum estimation for a G8 type star.

Figure 3 shows the estimation of the local continuum using the final method for an intermediate spectrum. The automatic adjustment is shown in blue, while the continuum estimated by the experts in each case is shown in red. As can be seen, the estimation is generally quite correct, but in some areas (marked in green) there are deviations from the estimation made manually by experts; in some regions where there is a lot noise or a large profusion of spectral lines, the signal does not smooth properly, since the computational algorithm includes more peaks than desired, resulting in higher values than the actual value estimated by the experts.

### 2.4. Artificial Neural Networks

Neural networks are an ideal technique for solving classification problems, especially for their ability to learn by induction and their capability to discover the intrinsic relationships that underlie the data processed during their learning (training phase). One of the major advantages of this AI technique is its generalization capability, which means that a trained network could classify data of a similar nature to those in the training set without any previous presentation. 

At this point in our development, we again used spectra from the main guiding catalog, originating from the same libraries that were applied to design and implement the knowledge-based systems’ reasoning rules [4,5,29]. By following this design strategy, the applied techniques could easily be compared, since their design principles and bases are the same.

Although it is possible to use all the available data for the learning phase, our experience in previous works advise us to split the available spectra into three sets, dedicating approximately 50% of them to the training, 10% to validating the learning, and 40% to testing the implemented networks. In the design of some of the neural networks, the spectra of the secondary catalog have also been used, because they allow us to evaluate the behavior of the networks in a sample with a greater variety of spectral features; these spectra are real, not estimations, and are affected by factors such as interstellar reddening or atmosphere distortion effects. The detailed distribution of the spectra sets is shown in Table 2.

The spectral analyzer, designed according to the knowledge-based system approach, is equipped with functions that allow it to obtain automatically the training, testing, and validation patterns presented to the neural networks. In most cases, neural networks designed for spectral classification have been trained and tested primarily with input patterns that include the measurement of the 25 spectral features obtained after performing the sensitivity analysis (Indices 1–25 in Table 1). 

Since we did not previously select the relevant parameters per spectral type or luminosity class, most neural models that were implemented count 26 units in the input layer (25 for the selected indices and 1 additional neuron, the so-called teaching input, for the supervised training models). On the other hand, all networks that use spectra from the secondary catalog (Prugniel 2001) only have 16 input patterns, since this database does not cover the entire spectral range in which the 25 selected indices are located. However, in some cases, we have also implemented networks that use a subset of spectral characteristics, with the objective of analyzing the influence of different sets of parameters in obtaining the MK classification. Furthermore, in some particular designs, we have used input patterns representing the real spectral flux in specific regions of wavelengths, which means that full spectral areas are provided to the neural network so that it is capable of extracting the relevant information on its own.

We started our development by scaling the spectra of every catalog to 100 at wavelength 5450 Å to obtain normalized values that are adapted to fluxes of the reference catalogs. We then calculated the value of the 25 spectral parameters in order to build the pattern sets. There was no need to cover the full spectral range, merely the range 3900–7150 Å, which is why the applied template spectra cover several spectral ranges. It is perfectly possible for the sampling frequency to differ according to the catalog. What happens is that the analyzer searches the lines and bands in a determined spectral area and then calculates the catalog resolution by means of a computational algorithm.

As soon as the spectral analyzer has gathered the input values, these values should be normalized and presented to the neural networks. The inputs were standardized by means of a contextualized normalization, which allows us to normalize the values in the [0, 1] interval and adequately scale and center each parameter’s distribution function. We assigned a lowest (*X_1_*) and a highest value (*X_2_*) to each classification index, establishing that 95% of the values lie between these two values; this provides us with constants *a* and *b*, which can be determined for each spectral parameter, according to values *X_1_* and *X_2_*:

(2) $$\begin{array}{c} 0.025=\frac{1}{1+e^{-\left(aX_{1}+b\right)}}\\ 0.975=\frac{1}{1+e^{-\left(aX_{2}+b\right)}} \end{array}.$$

At the present day, there are many models of artificial neural networks with different design, learning rules, and outputs. We have selected different architectures to address the automatic classification of optical spectra, carrying out the training process with both supervised and unsupervised learning algorithms. 

In particular, we have designed backpropagation networks (multi-layer architecture with supervised learning), SOM networks (self-organized maps with grid architecture and unsupervised learning) [24,25,33,34], and RBF networks (architecture in layers and hybrid learning) [35]. We have also analyzed some variants of these general network types (e.g., BP momentum), to decide the networks with a best performance in determining the MK classification. In the same way, we have studied each network behavior when using the different possible topologies (number of layers, nodes of the intermediate layers, etc.). As a preliminary step, we analyzed the ability of each of these three models to discriminate between consecutive types individually, corresponding to early, intermediate, and late stars. This initial experimentation certainly makes it easier to determine which network best fits each couple of successive spectral types.

We have used the SNNS v4.3 simulator (Stuttgart Neural Network Simulator) [36] for the design and training of classification networks. This software tool incorporates an option to convert directly the trained networks to C-code, which has been used in our work to incorporate the implemented networks into the global analysis and classification system for stars.

#### 2.4.1. Learning Algorithms

Our research has applied three different backpropagation (BP) learning algorithms for its initial developments: standard backpropagation, enhanced backpropagation (with a momentum term and flat spot elimination), and batch backpropagation (the weight changes are summed over each full presentation of all the training patterns).

In a first phase of experimentation, we have designed neural networks using the above-mentioned BP algorithms for the spectral types from B to M (type O was previously discarded due to its lack of presence in the reference catalogs). As for topology, classification networks have an input layer with 25 neurons (1 per each spectral index), one or more hidden layers, and an output layer with six units (1 for each MK spectral type). Since the backpropagation learning algorithm is a supervised one, it is necessary to provide the network with an extra unit (the teaching input) for each input pattern so that it can calculate the error vector and appropriately update the synaptic weights of its connections. 

In simulated systems, the most commonly used design strategy for configuring intermediate layers is to try to simplify them as much as possible, including the smallest number of neurons in each hidden layer, since each extra layer would involve a higher processing load in the software simulation. Thus, following this strategy, we have designed networks with different topologies, starting with simple structures and progressively increasing both the number of hidden layers and the number of units included in them (25 × 3 × 6, 25 × 5 × 6, 25 × 10 × 6, 25 × 2 × 2 × 6, 25 × 5 × 3 × 6, 25 × 5 × 5 × 6, 25 × 10 × 10 × 6, 25 × 3 × 2 × 1 × 6, 25 × 5 × 3 × 2 × 6, and 25 × 10 × 5 × 3 × 6). During the training, we updated the weights in topological order (input, hidden, and output layers). We opted for a random initiation of the weights with values from −1 to 1. We also modified the total training cycles, the validation frequency, and the learning parameters values (η, μ and flat) through the learning stage of the various implemented topologies.

Kohonen self-organized map unsupervised networks are unique in that they build preservation maps of the training data topology, where semantic information is transmitted by the location of a unit [33]. 

With a similar strategy to that of feed-forward nets, we have tested several Kohonen maps by grouping the MK spectral types into the same three sets (B-A, F-G, K-M). The SOM neural models do not validate the learning progress, so the validation patterns were added to the training dataset. In these networks, we have deliberately introduced another spectral type for every pair of consecutive types, in order to make it easier for the network to cluster the data more efficiently, contrasting the target spectra with very different ones in terms of morphology. Hence, this extra spectral type was selected to be as separate as possible from the spectra of each group, that is, we chose type M for early types and type B spectra for late and intermediate ones.

The input patterns of the Kohonen networks were also built by using the 25 essential spectral features shown in Table 1. In the learning phase of the designed networks, the total cycles and the learning parameters were adjusted (h(t), r(t), decrease factor for h(t), and decrease factor for r(t)).

The calculation of the chemical compositions or abundances of stellar spectra, and by extension MK classifications, could be also considered as a nonlinear approximation problem in which there are data affected by noise, and even the absence of data in the series occurs in some situations. The classical resolution strategy consists in performing a functional regression, which implies the determination of the specific function that best expresses the relation between the dependent and independent variables. In the case of RBF neural networks, it is not necessary to make any assumptions about the functions that relate outputs to inputs, since their basic principle is to center radial-based functions around the data that need to be approximated. Unlike MLP networks, the typical architecture of RBF networks is based on a feed-forward model composed strictly of three layers: an input layer with n neurons, a single hidden layer with k processing units, and a layer of output neurons [35].

We have designed RBF networks to obtain the different levels of classification in the MK system and, using again the simplification strategy of the network structure, we have considered the configurations 25 × 2 × 6, 25 × 4 × 6, 25 × 6 × 6, and 25 × 8 × 6.

After repeatedly trying the three described neural schemes for all the spectral types, we carefully chose the best network of each type so as to contrast their performance and determine which would be the best choice at the different scenarios of classification. The best implemented networks were studied by analyzing their results for the spectra of the test set.

The backpropagation (trained with enhanced learning algorithm) and RBF networks obtain a similar overall performance, 96% (0.96 ± 0.050) and 95% (0.95 ± 0.056), respectively (both intervals calculated for a statistical confidence level of 0.99). The BP network solves the problem better for some spectral types (F, K, M), whereas for other types (A) the RBF is a preferable option. However, the SOM maps obtained an inferior performance, about 68% (0.68 ± 0.117), in the best situations (networks trained with small decrease factors). These results are probably a consequence of the size and distribution of the training spectra, as these are not supervised networks and they need to make the clustering autonomously, so that they require a training dataset that is large enough to be able to abstract similarities and properly group the inputs.

As for the validation set, BP and RBF networks trained with a validation set of spectra present a higher success rate than the same networks trained without it. It is clear that the cross-validation technique influences the weights and allows the network to correct the weights dynamically during the training process and, therefore, improve the learning process.

Finally, the different topologies that were tested for each supervised learning algorithm (feed-forward and RBF) do not significantly affect the final performance of the network. However, we obtained the best results with a 25 × 5 × 3 × 6 backpropagation momentum network and with a 25 × 4 × 6 RBF network. SOM networks with a higher number of units in their competitive layer present a higher success rate; in fact, the networks designed with few units in the competitive layer (fewer than 12) are not capable of clustering the data properly.

Once we know the network with the best performance for each pair of spectral types, we are better able to propose neural models to undertake the complete MK classification process.

#### 2.4.2. ANN Models for Two-Dimensional MK Classification

The whole MK spectral classification includes the estimation of the spectral subtype and the luminosity class of the stars. Therefore, to carry it out completely, we here propose the implementation of several neural models based on two different design philosophies: classification based on a global network and classification structured in levels.

The first method proposed to obtain the whole MK classification is based on the design of two global artificial neural networks that estimate simultaneously and independently the spectral subtype and the luminosity class of the stars. 

**Figure 4:**
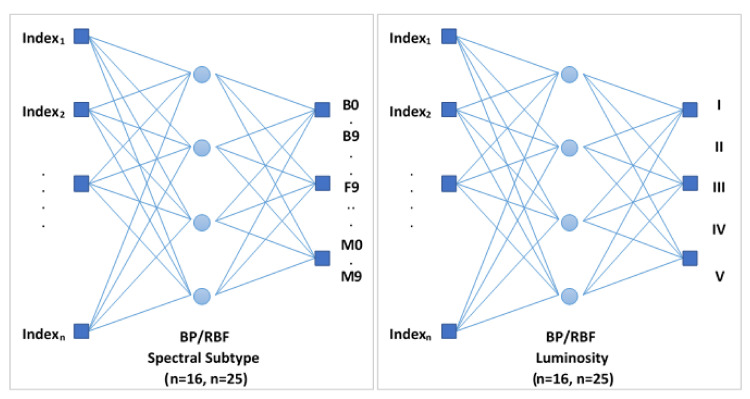
Conceptual scheme of the global networks designed for the complete MK classification.

In this way, this global networks include only BP and RBF neural structures, since SOM architectures were discarded because they obtained more modest results during previous experimentation. In the design of the networks, we implemented models that only support the 16 spectral indices present in the range 4100–6800 Å of the secondary catalog, and another alternative networks that accept as input the 25 spectral parameters of the full range (3510–7427 Å, corresponding to the main guiding catalog). Figure 4 illustrates the conceptual design of this first proposed model.

Our second strategy to accomplish the whole MK classification is based on a combination of the networks described in the previous sections, properly interconnected; specifically, we use those that have obtained a higher performance for each level of spectral classification (global, spectral type, spectral subtype, and luminosity) during the previous evaluation phase of the different neural configurations. These networks are now organized in a tree-like composition similar to the reasoning scheme of the previous designed knowledge-based systems [29].

In this way, BP (specifically networks trained with the backpropagation momentum algorithm) and RBF are implemented again, and they also accept as input both the 25 spectral indices belonging to the range of the main reference catalog and the 16 included in the most restrictive range of the secondary catalog.

Regardless of the learning algorithm and the spectral range, the MK classification net of networks is formed by a first level BP/RBF network that decides the global classification, that is early, intermediate, or late star; a second level made up by three different networks that classify each previous pair of spectral types; and a third level formed by twelve networks, two for each spectral type (B, A, F, G, K, and M), so that one of them is responsible for establishing the specific subtype (0–9) and the other one appropriately determines the luminosity class (I–V) depending on the specific estimated spectral type.

**Figure 5:**
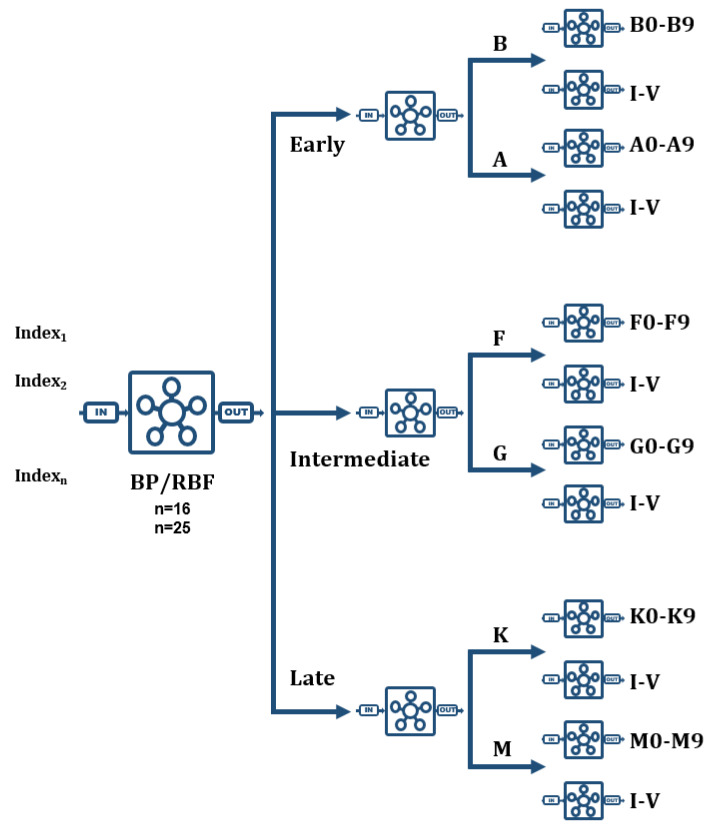
Conceptual scheme of the net of networks designed for the complete MK classification.

When implementing this tree structure in C++, the communication between the different classification levels was arranged appropriately, so that the networks at each level can decide which network in the next level send the spectra to and direct them towards it accompanied by a partial classification and a probability estimation. In some specific cases, it will be necessary to send them to two different networks, especially with spectra at the boundary between two consecutive spectral types, for example B9A0 or G9K0. Figure 5 shows a conceptual scheme of this second strategy.

Table 3 lists the different topologies selected in the two alternative strategies formulated to obtain the complete MK classification, as well as their performance for test spectra.

The classification results obtained with the different implemented methods will be now shown through the interface of the spectral analysis application. In the case of neural networks, besides the two-dimensional MK classification in spectral subtype and luminosity class, we also provide a probability that somehow informs about the quality of the offered answers, i.e., the network confidence in its own conclusions. In those cases, where the network output is ambiguous, falling within the range of non-resolution ([0.45–0.55]), two alternative classifications will be provided, each of them supported by their corresponding probability.

### 2.5. Final Approach: Hybrid System

The increasing complexity and sophistication of modern information systems clearly indicates that it is necessary to study the suitability of each technique at our disposal to develop adequate and efficient solutions, as we have done in the analysis presented in this paper. 

The hybrid strategies are mainly based on the integration of different intelligent methods (fuzzy logic, evolutionary computation, artificial neural networks, knowledge-based systems, genetic algorithms, probabilistic computation, chaos theory, automatic learning, etc.), in a single robust system that explores their complementarities with the objective of achieving an optimal result in the resolution of a specific problem [37]. In this way, hybrid systems combine different methods of knowledge representation and reasoning strategies in order to overcome the possible limitations of their integrated individual AI techniques, and increase their performance in terms of exploiting all available knowledge in an application field (symbolic, numerical, inaccurate, inexact, etc.) and, ultimately, offer a more powerful search for answers and/or solutions.

Although there are several possibilities for the integration and cooperation between the different components of the hybrid systems, the conclusions of our previous experience with the different individual techniques, naturally point to a hybrid modular architecture for the automation of the MK classification. Our proposal would be made up of both symbolic (knowledge-based systems) and connectionist (neural networks) components, combined in a single functional hybrid system that operates in co-processing mode. 

The methods with a more satisfactory performance during the previous experimentation stage were directly transferred from their respective development environments to a single hybrid system developed in language C ++. 

In this global system, it will now be possible to perform the spectral classification from two different perspectives: separately with each of the individual techniques, obtaining a set of isolated predictions that the user will be responsible for analyzing to arrive at a compromise solution; or initiating a combined classification process that automatically invokes the most efficient methods of each implemented technique (expert systems, backpropagation net of networks, RBF global network, etc.) and finally presents the user a set of answers accompanied by an associated probability, making possible a quick evaluation of the conclusions quality. In this second classification strategy, the system itself is responsible for analyzing the results of each technique, grouping them together and providing a summary, so that it is possible to offer better conclusions at the same time that we grant the automatic procedure of some degree of self-explanation.

Based on the experience acquired during the study phase, the experts assigned to each different technique a set of probability values according to its resolution capacity in the classification of the spectra belonging to each spectral type or luminosity class. Then, when the hybrid strategy is launched, the different optimal classifiers must combine the probability of their respective conclusions (derived from the stellar classification process) with the numerical value that they have associated by default, and which is indicative of the grade of confidence of their estimations for a specific spectral type or luminosity class. 

**Figure 6:**
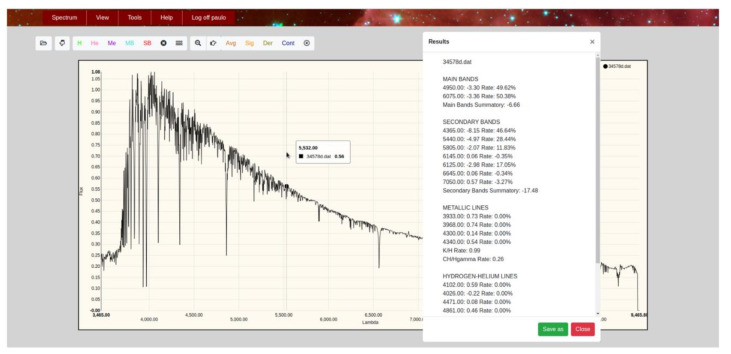
Hybrid system interface showing the combined classification with optimal methods.

Figure 6 shows in the hybrid system interface the results obtained in a specific classification assumption when selecting the option based on the described hybrid approach.

The integration of the two described types of classification into a single hybrid system allows this computational implementation to emulate, in a more satisfactory way, the classical methods of analysis and classification of stellar spectra that we describe in previous sections. The option that combines the optimal implementations of each technique obtains a classification based on solid foundations that result in clear and resumed conclusions for each classification level. At the same time, it is possible to carry out a detailed analysis of each individual spectrum with all the methods implemented and to study the discrepancies or coincidences between the estimations provided by each of them.

## 3. Results

To accomplish a complete analysis of the performance achieved with the different computational techniques, we now study their results in the spectra of the test datasets from both the main and the secondary reference catalogs. It is important to remark that these sets had not been previously used in the design of the expert systems or in the training of the different neural networks.

Every method processes and analyzes the spectra and obtains a two-dimensional classification in the MK system (spectral subtype, indicative of the surface temperature, and stellar luminosity), both accompanied by an estimation of the confidence in the classification (in form of probability). In the analysis of results, we do not consider as correct classifications the conclusions whose probability or confidence does not exceed 70%. In addition, in the study of the outputs of the subtype networks, the procedure for evaluating the successes is similar to that carried out by most experts when they compare their classifications, since we accept a resolution of ±1 spectral subtypes. In this sense, if the network places the subtype within the correct group, the result is computed as valid; otherwise, the two adjacent subtypes are examined (higher and lower temperatures), and the result would be considered correct if the output belongs to one of these subtypes. Otherwise, it would be computed as a real error. Regarding luminosity, as most of expert spectroscopists do, we consider that the valid outputs are only those where the class estimated by the model exactly matches the one specified in the reference catalog.

**Figure 7:**
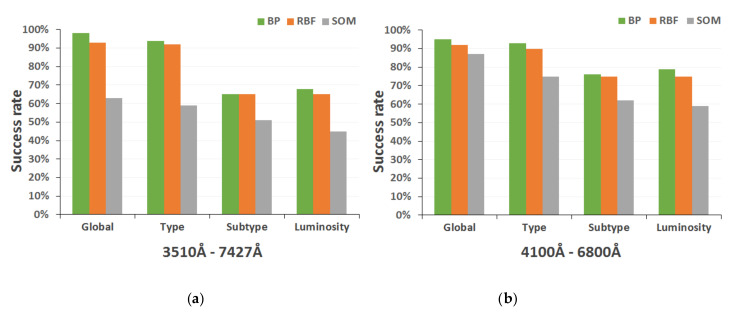
Performance analysis of classification neural network architectures: (**a**) spectral range 3510–7427 Å; and (**b**) spectral range 4100–6800 Å.

Figure 7 shows the comparative performance analysis of the three neural architectures finally selected (BP momentum networks, RBF networks, and SOM maps) for each classification level (global, spectral type, spectral subtype, and luminosity class) and each spectral range.

Table 4 shows the confusion matrix [38] for the spectral type corresponding to the analysis of the outputs of the neural model with optimal results, that is, the net of networks trained with the backpropagation momentum learning algorithm. In the rows of this useful graphical tool, we include the actual number of spectra of each spectral type, following the catalog classifications, and, in the columns, we show the number of predictions obtained for each of them with our neural implementation.

By examining the resulting matrix, it becomes clear that errors generally occur between contiguous types, that is, which presumably is due to the edge problem already mentioned in previous sections (errors in subtypes that fall on the boundaries between two different spectral types, such as B9A0 or K9M0). Therefore, if we use the classical resolution of ±1 spectral subtypes in the analysis of the outputs, the resulting confusion matrix (second numbers of each cell included in Table 4) shows a considerable decrease in classification errors.

**Figure 8:**
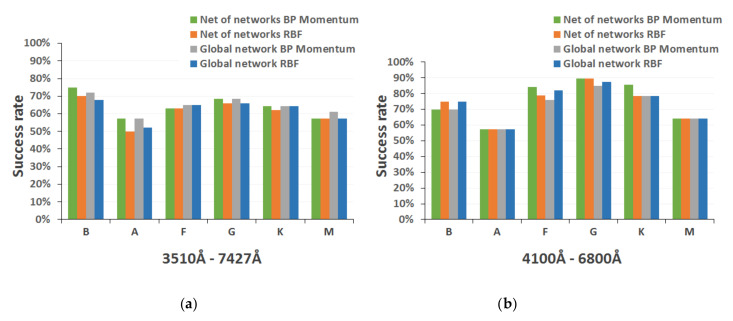
Performance analysis of neural models for final MK spectral type classification: (**a**) spectral range 3510–7427 Å; and (**b**) spectral range 4100–6800 Å.

**Figure 9:**
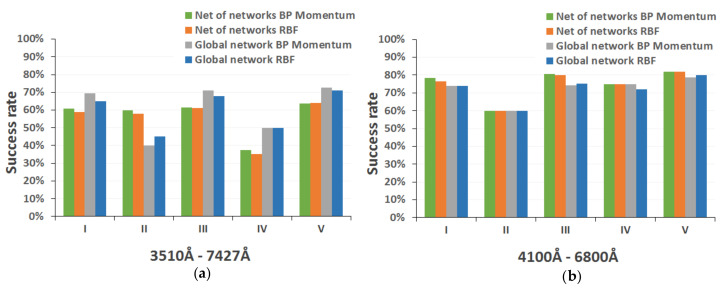
Performance analysis of neural models for final MK luminosity classification: (**a**) spectral range 3510–7427 Å; and (**b**) spectral range 4100–6800 Å.

Figure 8 shows the performance comparison of the two proposed strategies for the entire MK classification, detailed by spectral type, again using test spectra in both catalog spectral ranges. Figure 9 presents the homologous performance comparison graphs for luminosity level.

According to the results obtained with the different neural models implemented for the complete MK classification, we considered that it would be convenient to include in our final system the net of neural networks for the determination of both the spectral subtype and the luminosity class, since the success rates are satisfactory in the two dimensions (around 75%, 0.75 ± 0.049 for a statistical confidence level of 0.99) and slightly higher than those achieved by the specific global network (about 73%, 0.73 ± 0.051 for a statistical confidence level of 0.99).

**Figure 10:**
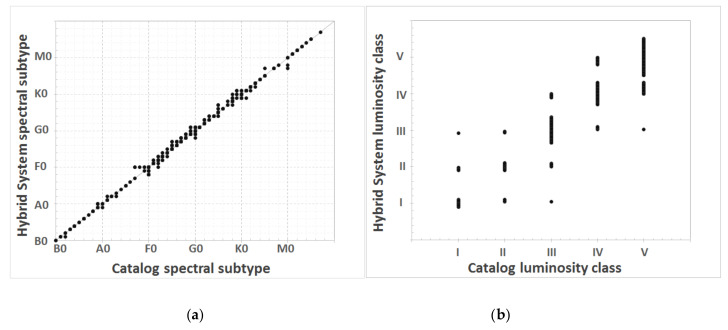
Comparison of the MK classifications obtained by the hybrid final system with the reference catalog stellar classifications: (**a**) spectral type; and (**b**) luminosity class.

As mentioned in Section 2.5, the final classifier that we propose is a hybrid system that combines the methods that resulted in better performance during our previous experimentation stages. Figure 10 shows the results for both MK system dimensions for the whole test set of 600 spectra, versus the actual classification provided by the guiding catalogs for these spectra.

Table 5 shows the mean absolute errors (in black color) and standard deviations (in blue) for the complete MK classification, which were obtained with our hybrid system using again the 600 test spectra.

To complement the evaluation of the results, we carried out an additional validation of the optimal individual models and the proposed hybrid system, also contrasting their conclusions with the classifications obtained by the group of expert spectroscopists of our project. In this final study, we again consider the two sets of test spectra, which correspond to 600 spectra in both the spectral range 3510–7427 Å and the spectral range 4100–6800 Å.

**Figure 11:**
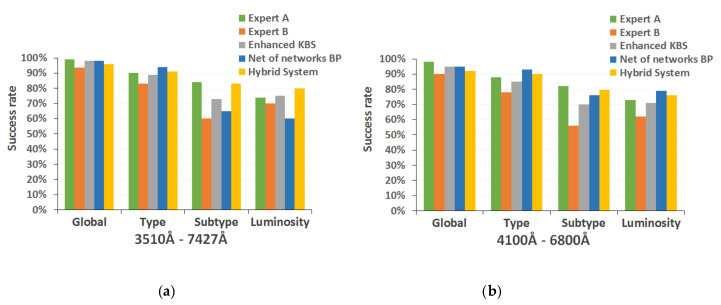
Final performance analysis comparing hybrid model with each individual technique: (**a**) spectral range 3510–7427 Å; and (**b**) spectral range 4100–6800 Å.

Figure 11 shows the success rates obtained by the global hybrid system and each of the individual artificial intelligence methods in the complete classification of the 600 test spectra, for both spectral ranges.

Table 6 shows, as a percentage of agreement, the results of a double-blind study that consists of evaluating the correctness of the classifications assigned to the spectra without knowing their origin. On the one hand, the estimations obtained with the different computational techniques were compared with those of the human experts who collaborate in the development of this system. On the other hand, the spectroscopists of our group analyzed again the spectra already classified by the different automatic techniques, expressing their agreement or disagreement with the classifications concluded by each one (highlighted in Table 6).

**Figure 12:**
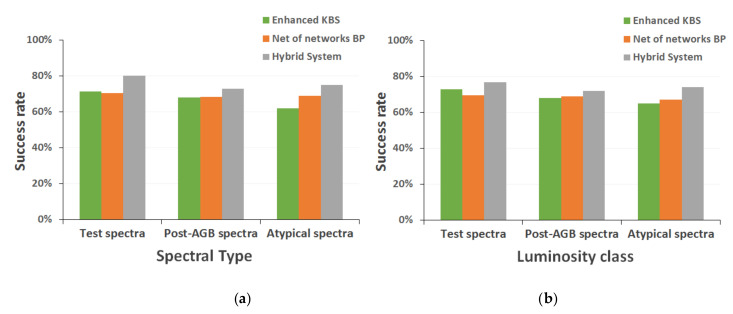
Results with different sets of peculiar spectra (**a**) spectral type; and (**b**) luminosity class.

Finally, Figure 12 shows the performance of the best techniques when applied to the classification of larger and heterogeneous spectral sets, such as the one formed by 49 real post-AGB stars from our own observation campaigns [39] or the set composed by the 486 spectra initially discarded from all the reference catalogs for reasons such as an atypical metallicity level, lack of spectral fluxes in some areas, or an incomplete classification in the available sources.

## 4. Discussion

In our preliminary studies of the different selected neural models, we found (as the graphs in Figure 7 show), that the backpropagation and RBF networks obtain a very similar results (with some slight differences), whereas the Kohonen networks present significantly lower success rates in all implementations. As mentioned above, self-organized maps are mainly characterized by the use of a non-supervised and competitive learning algorithm, in which it is essential that the training set is large enough to allow the network to extract the similarities between the input data and cluster them appropriately. For this reason, the SOM networks trained with the spectra from the secondary catalog (Figure 7b) show a more adequate behavior, with success rates more similar to those obtained with the other neural models. It is clear that the addition of more spectra to the training set allows the creation of two-dimensional output maps more adapted to the problem of spectral classification.

Analyzing the results obtained during the previous experimentation phases, we can conclude that the neural architectures based on the multi-layer perceptron (BP) and on radial basis functions (RBF) have a more congruent behavior, being able to achieve a very acceptable final performance for the different classification levels. That is, for both spectral ranges, they reach success rates above 90% (0.9 ± 0.077 for a statistical confidence level of 0.99) in the preliminary discrimination and in the determination of the spectral type, and around 70% (0.7 ± 0.012, for a statistical confidence level of 0.99) in obtaining the subtype and luminosity class. For this reason, these architectures have been included in the implementation of our final developed system of spectral analysis and classification.

The detailed analysis of the performance achieved with the two design strategies for the full MK classification (shown in the comparative charts of Figure 8) indicates that, taking into account the results as a whole, the model based on a tree structure presents a more satisfactory success rate, since it is able to obtain correctly and unequivocally (probability greater than 70%) the spectral subtype of about 76% of the spectra in networks trained with the backpropagation momentum algorithm (0.76 ± 0.049, for a statistical confidence level of 0.99) and around 74% when they are trained with the RBF algorithm (0.74 ± 0.05, for a statistical confidence level of 0.99). By contrast, in implementations based on the direct determination of the subtype by means of global networks, this performance is slightly lower (72–73%, 0.72 ± 0.052 for a statistical confidence level of 0.99). The inclusion of spectra from the secondary catalog (Figure 8b) generally produces a significant improvement in the performance of each of the models implemented (between 7% and 13%).

Thus, for the first MK dimension (the spectral type), the study of the results denotes that there could be a certain correlation between the final success rate and the composition and size of the training set, since the networks performance decreases for the types in which that set includes less spectra (A and M, mainly), regardless of the model and the spectral range. When the spectra of the secondary catalog are introduced, the results improve significantly (by more than 20% in some cases) for the intermediate types (F, G, and K), because in these types the catalog presents a greater increase in both the number and the variety of spectra. On the other hand, for the other spectral types (B, A, and M), the performance does not increase noticeably and sometimes even decreases, since the stars of these types are more sensitive to the spectral indices that have been excluded from the secondary catalog range (mainly some important lines of hydrogen and helium around 4000 Å and some late bands of TiO in the region of 7000 Å).

To continue the study of the results for the MK spectral type, we built the confusion matrix of the BP net of networks, which is the architecture that achieved the best performance. In this case, the neural model correctly assigns the spectral type to a total of 83 test spectra (shown in the main diagonal in Table 4), which implies a success rate of 83% (0.83 ± 0.096, for a statistical confidence level of 0.99) with a mean error of 0.2 types. A detailed observation of the matrix shows that most errors takes place between adjacent types, which is consistent with the typical problem concerning to the subtypes that are located in the limits between spectral types, already mentioned in previous sections. For this reason and with the aim of getting closer to the real analysis carried out by the experts, we decided to rebuild the matrix using the resolution of ±1 spectral subtypes that human experts normally use (shown in the second numbers of each cell in Table 4). As expected, the percentage of errors decrease with this adaptation, since the network is now capable of assigning a spectral type congruent with the one that gives their own catalog to 93 of the 100 evaluation spectra (0.93 ± 0.065, for a statistical confidence level of 0.99), reducing the mean error to 0.08. Although the success rate increases by 10%, in this new matrix, there is still some confusion between spectral types A and F (highlighted in Table 4). These errors in classification are in some way predictable, since these spectral types are usually affected by the classic degeneracy problem between the surface temperature of the star and the metallicity of its spectrum.

The exploration of the results of the other MK dimension, luminosity (Figure 9), shows that both implemented final models reach a similar performance (60–68%) for the spectral range of the main catalog, although the option based on specific networks of luminosity shows a slight improvement (global network approach). This can be due to the additional errors that are included in the net of networks model when the spectral type is determined by following a wrong path in the tree, since it adds an extra and prior error that conditions the further estimation of the luminosity class. However, this trend is reversed by adding the spectra from the secondary catalog, since the model based on a net of networks (composed of several consecutive neural networks that obtain the luminosity after having determined the spectral type) achieves an optimal performance. They estimate adequately (probability greater than 70%) the luminosity class of 79% (0.79 ± 0.047, for a statistical confidence level of 0.99) of the spectra for backpropagation networks and around 75% (0.75 ± 0.05, for a statistical confidence level of 0.99) for RBF networks.

As in the spectral type, if we analyze the results considering the level of luminosity, we can note again a clear dependence on the size of the training set, since the performance decreases significantly in those luminosity levels where this set is scarcer (II and IV), independently of the neural model and the spectral range. Obviously, the more spectra are available for each class, the better is the delimitation of the spectral features that define it, so that the obtained results would be perfectly consistent with the design of the neural models. For this reason, when secondary spectra are added, performance tends to improve for all classes but especially for the less favored ones, where the sample size was significantly smaller, reaching an increase of more than 25% in some implementations. Similarly, the highest success rate is achieved for the luminosity V, because the number of patterns that belong to this class is significantly higher, with more than 200 spectra of difference in some cases.

The detailed analysis of the performance achieved in the two dimensions of the MK classification, led us to finally use the strategy based on a net of neural networks for both the spectral subtype and the luminosity class, since it presents sufficiently adequate success rates in both dimensions (about 75%, 0.75 ± 0.05 for a statistical confidence level of 0.99) and somewhat better than those obtained with the strategy of global networks (73%, 0.73 ± 0.051 for a statistical confidence level of 0.99). As we could already see in the sensitivity analysis carried out on spectral classification indices (Table 1), many of the parameters used by experts affect temperature and stellar luminosity at the same time, and it is extremely difficult to separate both influences. Therefore, a neural model based on a multi-level classification that determines the luminosity class as a function of the previous spectral type estimation will also be more consistent with the deduction process followed in the classical stellar classification techniques.

The next phase of the results analysis was the evaluation of the hybrid model proposed as a final implementation of the classification system (Figure 10 and Table 5), also analyzing its behavior compared to the other previously implemented classification methods (Figure 11). We also carried out a double-blind study to validate the correction of the classifications without knowing their source; in this way, the classifications assigned by the human experts who collaborate in this project were compared with those obtained with the different computational techniques (Table 6). As an additional test, we studied the performance of the implemented methods when applied to larger, heterogeneous, and unusual spectral sets (Figure 12), i.e., real stars of our observation campaigns, spectra with peculiarities, etc. 

As previously, the errors in some spectral types such as A and F appear again with the hybrid system, which can be clearly seen in the graph in Figure 10, where a greater dispersion with respect to the diagonal (perfect classification) can be observed in the regions corresponding to the subtypes of these spectral types. Likewise, these types present a greater mean absolute error and a greater standard deviation. As for the other dimension, the luminosity classes II and IV are where the greatest errors continue to be concentrated.

The results obtained in these final comparative studies confirm the hypothesis that the hybrid approach proposed emulates the behavior of human experts in this field more satisfactorily than any of the individual computational techniques. The hybrid system’s success rate in discrimination of the spectral subtype, 80.16% (0.8016 ± 0.042, for a statistical confidence level of 0.99) with an absolute mean error of ±0.25 subtypes and a standard deviation of 0.54, and the level of luminosity, 76.83% (0.7683 ± 0.044, for a statistical confidence level of 0.99) with an absolute mean error of ±0.24 classes and a standard deviation of 0.44, is superior (even for the most refined versions of individual methods) and it is maintained at a higher level when determining the classification of broader sets of spectra that have special characteristics, such as a high noise level or an insufficient resolution (shown in graphs in Figure 12). In addition, the agreement between this hybrid system and the experts who participated in the blind study is also superior to the one presented with any other methods. 

This propensity towards performance improvement was somewhat expected, especially in view of the fact that the hybrid approach is essentially based on a classification strategy that selects the best conclusions from the optimal methods according to a heuristic estimation of the confidence level of the predictions. Thus, it is possible to mitigate some of the problems that individual methods have proved unable to manage, such as the confusion between stars B and M in cases where emission occurs instead of absorption. However, other problems do not find solution in this alternative either, for example the classical confusion between spectra A and F of low metallicity level.

## 5. Conclusions

In this paper, we propose a detailed analysis of how knowledge-based systems and ANNs can be applied to the MK classification of stellar spectra. If knowledge-based systems are found most appropriate for global and generic classifications, neural networks are shown to adequately establish the stellar spectral types and luminosity.

Following the classic method of manual classification, we built two different catalogs of spectra covering the whole range of MK spectral types and luminosity classes. The spectra of these databases were used to define the rules of expert systems and to train and validate the artificial neural networks.

Since human experts visually study certain spectral features, we defined an initial set of 33 spectral indices that characterizes each spectrum and reduces its processing complexity. This first set was refined by performing an exhaustive sensitivity analysis that helped us to determine the actual classification capacity of each of the proposed indices. As a result, we obtained a final set of 25 spectral features that were used both to define the fuzzy sets in TKS expert systems and to build the input patterns of neural networks.

The paper describes our complete experimentation with various ANN models and analyzes their performance and results. Thus, we designed and tested BP, SOM, and RBF networks, and checked a variety of topologies, which can provide the stars’ MK classification in three levels (global, spectral type and subtype, and luminosity class). We established that the finest networks could determine the spectral type in a sample of 100 testing spectra with an accuracy of almost 95% (0.95 ± 0.056, for a statistical confidence level of 0.99).

To accomplish the whole two-dimensional MK classification of stars, we have adopted two neural strategies that essentially differ in their structure: global approach and nets of networks. According to the results obtained, we can conclude that it is more efficient to use a net of neural networks for the determination of the spectral subtype and the luminosity class, since the success rates are satisfactory enough in both dimensions (around 75%, 0.75 ± 0.05 for a statistical confidence level of 0.99) and also slightly higher than those obtained with the specific global network (around 73%, 0.73 ± 0.051 for a statistical confidence level of 0.99). 

In our final proposal, all the computational techniques were combined into a hybrid system that decides the proper procedure for the different classification contexts, therefore reaching greater adaptation to the problem than a classification tool based on a single technique. The obtained results suggest that the hybrid strategy is a more adequate and versatile approach, since it achieves higher success rates than any other individual method (around 80%, 0.80 ± 0.042 for a statistical confidence level of 0.99).

Our most recent research work consists in the analysis of functional networks, to establish whether they are apt for stellar classification, and in the completion of our stellar database and its adaptation to the Internet. We want to make our database accessible so that users all over the world may store and classify spectra and as such contribute to making our automatic analysis and classification system more adaptable and accurate.

Gaia’s DPAC, to which our research group belongs, is currently developing several software modules to approach stellar parameterization by the use of RVS instrument data from different perspectives [40]. A second step will be the inclusion of a MK classifier such as the one presented here, only for late and intermediate type stars, adapted to the Gaia RVS spectral region. We started the work based on simulations, although in recent months we dispose of real data for algorithms validation (under NDA, not publishable results). Some data and public results of the WP will be publicly available in Data Release 3 (DR3) at the end of 2021 and as such will constitute a hard big data environment (more than 1 Petabyte of data) for some of the techniques presented here.

## Figures and Tables

**Table 1:** Sensitivity analysis results for the selected classification indices.

Index	Theoretical Resolution	Actual Resolution
1. B (4950 Å)	Global ^1^	K-M types Luminosity I for F-G, Luminosity III for M
2. B (6225 Å)	Global ^1^	G-M types, K-M subtypes Luminosity I for G-K, Luminosity V for K-M
3. B (5160 Å)	K-M types	G-M types
4. B (5840 Å)	K-M types	K-M subtypes
5. B (5940 Å)	K-M types	M subtypes
6. B (6245 Å)	K-M types	K-M types
7. B (6262 Å)*	K-M types	K-M types
8. B (6745 Å)	K-M types	M subtypes Luminosity III-V for M
9. B (7100 Å)*	K-M types	M subtypes
10. I (3933 Å)	A-G types	F-G subtypes Luminosity I for B-F
11. I (3968 Å)	A-G types	A-M types ^2^Luminosity I for B-F
12. I (4340 Å)	B-G types Luminosity I for early	A-G subtypes ^2^ Luminosity I for B-A
13. I (4102 Å)	B-G types Luminosity I for early	A-F subtypes
14. I (4026 Å)	O-B types	B type
15. I (4471 Å)	O-B types	B type
16. I (4861 Å)	B-G types Luminosity for early	B-K types, A-G subtypes ^3^ Luminosity for B-A
17. I (6563 Å)	B-G types Luminosity I for early Spectra with emission	B-K types^1^, A subtypes
18. EW (4300 Å)	A-G types	A-G types, F-G subtypes Luminosity I for K-M
19. I (3933 Å)/I (3968 Å)	A-G types	A-F types
20. I (4102 Å)/I (4026 Å)	B-A types	B type
21. I (4102 Å)/I (4471 Å)	O-A types	B subtypes Luminosity for B
22. EW (4300Å)/I (4340Å)	F- K types	G-K types
23. ∑_1,2_Bi*	Not included	Global ^1^ F-M types, G-M subtypes Luminosity I for F-K, Luminosity V for K-M
24. ∑_3..9_Bi*	Not included	K-M subtypes
25. ∫I(λ_i_) dλ*	Not included	Early, intermediate
26. B (4953 Å)	K-M types	Not valid (value only from M5)
27. B (6140 Å)	K-M types	Not valid (value only from M4)
28. B (4435 Å)	K-M types	Not valid (includes other indices)
29. B (5622 Å)	K-M types	Not valid (arbitrary behavior for B-M)
30. B (5805 Å)	K-M types	Not valid (includes other indices)
31. I (4144 Å)	O-B types	Not valid (wrong behavior for M)
32. I (4481 Å)/I (4385 Å)	F-G types	Not valid (arbitrary behavior for B, F)
33. I (4045 Å)/I (4173 Å)	A-G types	Not valid (arbitrary behavior for F-M)

^1^ Early, Intermediate, Late. ^2^ With previous global classification. ^3^ With previous type classification. *B* means the depth of the molecular bands, *I* is the intensity, and *EW* is the equivalent width of the absorption/emission lines

**Table 2:** Datasets for artificial neural networks, detailed by spectral type and luminosity class.

MK Spectral Type	Training Set	Validation Set	Test Set
B	25	5	20
A	20	3	14
F	24	5	19
G	25	5	19
K	20	3	14
M	20	3	14
TOTAL	134	24	100
**MK Luminosity**	**Training Set**	**Validation Set**	**Test Set**
I	30	6	23
II	10	1	5
III	40	7	31
IV	11	2	8
V	43	8	33
TOTAL	134	24	100

**Table 3:** Implemented neural architectures for complete MK classification.

Network Type	Spectral Range	Topology (Success Rate)
Net of networks BP Momentum	3510 Å–7427 Å	Global: 25 × 10 × 3 (98%); Type: 25 × 5 × 3 × 2 (94%) Sub.: 25 × 20 × 20 × 10 (65%); Lum.: 25 × 10 × 10 × 5 (60%)
Net of networks BP Momentum	4100 Å–6800 Å	Global: 16 × 10 × 3 (95%); Type: 16 × 10 × 5 × 2 (93%) Sub.: 16 × 50 × 20 × 10 (76%); Lum.: 16 × 20 × 10 × 5 (79%)
Net of networks RBF	3510 Å–7427 Å	Global: 25 × 15 × 3 (93%); Type: 25 × 4 × 2 (92%); Sub.:25 × 20 × 10 (62%); Lum.: 25 × 20 × 5 (60%)
Net of networks RBF	4100 Å–6800 Å	Global: 16 × 15 × 3 (92%); Type: 16 × 4 × 2 Sub.: 16 × 25 × 10 (75%); Lum.: 16 × 30 × 5 (75%)
Global network BP Momentum	3510 Å–7427 Å	Sub.: 25 × 30 × 20 × 10 (67%) Lum.: 25 × 10 × 10 × 5 (68%)
Global network BP Momentum	4100 Å–6800 Å	Sub.: 16 × 50 × 20 × 10 (74%) Lum.: 16 × 20 × 10 × 5 (75%)
Global network RBF	3510 Å–7427 Å	Sub.: 25 × 30 × 10 (65%) Lum.: 25 × 20 × 5 (65%)
Global network RBF	4100 Å–6800 Å	Sub.: 16 × 30 × 10 (75%) Lum.: 16 × 30 × 5 (73%)

**Table 4:** Initial and final confusion matrix for the spectral type in the optimal neural model.

Real/Estimated	B	A	F	G	K	M
**B**	19/20	1/0	0/0	0/0	0/0	0/0
**A**	1/1	10/11	3/2	0/0	0/0	0/0
**F**	0/0	**4/2**	**14/17**	1/0	0/0	0/0
**G**	0/0	0/0	1/1	16/18	2/0	0/0
**K**	0/0	0/0	0/0	1/0	12/14	1/0
**M**	0/0	0/0	0/0	1/1	1/0	12/13

**Table 5:** Mean absolute errors and standard deviations for system classifications by spectral type.

	All	B	A	F	G	K	M
**Spectral type**	0.25/0.54	0.13/0.33	0.50/0.91	0.29/0.56	0.23/0.50	0.15/0.42	0.24/0.77
**Luminosity**	0.24/0.44	0.10/0.30	0.35/0.56	0.31/0.47	0.24/0.44	0.10/0.30	0.05/0.22
**Number of spectra**	600	40	26	241	180	92	21

**Table 6:** Double blind study comparing human experts and computational sources.

	Expert A	Expert B	Fuzzy KBS	BP Networks	Hybrid System
**Expert A**	**100%**	**78%**	**86%**	**85%**	**89%**
**Expert B**	**87%**	**100%**	**86%**	**86%**	**83%**
**Fuzzy KBS**	84%	76%	100%	79%	78%
**BP networks**	84%	78%	79%	100%	75%
**Hybrid system**	85%	72%	78%	75%	100%
